# Effects of Different Repetitive Transcranial Magnetic Stimulation Targets for Enhancing Motor Learning: A Single‐Blind Randomized Controlled Trial

**DOI:** 10.1155/np/6487874

**Published:** 2026-06-04

**Authors:** Alfredo Lerín-Calvo, Marcos Moreno-Verdú, Lorena Remón-Ramiro, Robert M. Hardwick, Carlota Lasheras-Cuerda, Sergio Lerma-Lara, Raúl Ferrer-Peña

**Affiliations:** ^1^ Applied Neurosciences Research Group in Rehabilitation (GINARE), Neuron, Madrid, Spain; ^2^ Department of Physiotherapy, CSEU La Salle, Autonomous University of Madrid, Madrid, Spain, uam.es; ^3^ Clinical-Educational Research Group on Rehabilitation Sciences (INDOCLIN), CSEU La Salle, Autonomous University of Madrid, Madrid, Spain, uam.es; ^4^ Brain, Action and Skill Laboratory (BAS-Lab), Institute of Neuroscience (Cognition and Systems Division), UCLouvain, Louvain, Belgium

**Keywords:** healthy, motor learning, neuromodulation, primary somatosensory cortex, repetitive transcranial magnetic stimulation

## Abstract

**Background:**

The primary motor (M1) and somatosensory (S1) cortices play a key role in motor learning (ML), but the physiological mechanisms underlying learning in these regions remain unclear. This study aimed to evaluate the effect of high‐frequency repetitive transcranial magnetic stimulation (rTMS) applied over S1 or M1 to enhance use‐dependent and reinforcement‐like ML in healthy participants.

**Methods:**

A single‐blind, randomized controlled trial (RCT) was conducted with 60 healthy participants, randomly assigned to receive rTMS over M1, S1, or sham. Participants underwent a 10‐day motor training protocol involving an arbitrary visuomotor association (AVMA) task to promote reinforcement learning and the Purdue Pegboard Test (PPT) to promote use‐dependent learning, always after rTMS. The primary outcome measure was the number of pegs placed in the PPT, and the secondary outcome was the response accuracy during a forced response (FR) version of the AVMA. Assessments were conducted at baseline, mid‐intervention (*t*1), postintervention (*t*2), and at 1‐month follow‐up (*t*3).

**Results:**

For use‐dependent learning on the PPT, S1 stimulation outperformed sham at *t*1 (right hand), *t*2 (both hands), and *t*3 (both hands). M1 stimulation showed no significant differences versus sham. For reinforcement learning on the forced response‐AVMA (FR‐AVMA), no active group differed from sham; however, S1 and M1 showed opposite numerical tendencies at *t*3 (S1 trending better, M1 worse), yielding a significant S1–M1 contrast.

**Conclusion:**

These findings suggest that rTMS over S1 may enhance use‐dependent ML, while the divergent S1 and M1 tendencies in reinforcement‐like learning warrant further, adequately powered investigation, with implications for future research in both healthy and clinical populations.

**Trial Registration:**

ClinicalTrials.gov identifier: NCT06262425

## 1. Introduction

Motor learning (ML) is defined as “relatively permanent changes in the ability to perform specific activities or behaviors, resulting from practice or experience, measured through retention tasks” [1]. The acquisition of motor skills depends on two relatively separate neural networks: the associative/premotor (AP) network and the sensorimotor (SM) network [2].

The AP network includes the dorsolateral prefrontal cortex, premotor areas, supplementary motor area, inferior parietal cortex, cerebellum, and rostral basal ganglia, while the SM network comprises the primary somatosensory cortex (S1), primary motor cortex (M1), caudal basal ganglia, and cerebellum [2–5]. During ML, there is typically initially greater activity in the AP network, followed by increased activity in the SM network [5]. Previous meta‐analyses [6] have identified common areas involved in ML across different tasks, which were labeled as the “core” network of ML. The left dorsal premotor cortex, bilateral supplementary motor area, M1, S1, and thalamus were key structures heavily involved in ML.

The neural circuits involved in ML are not only time‐dependent but also vary depending on the type of motor task being learned and therefore the primary physiological mechanism(s) underlying improvements in that skill. To date, four processes have been described as potential mechanisms mediating ML: error‐based, use‐dependent, reinforcement, and strategy‐based learning [7]. Our study focuses on determining the relative contribution of S1 and M1 to two of these processes: use‐dependent and reinforcement learning, with the important caveat that the task employed likely recruits multiple, partially overlapping mechanisms rather than isolating a single learning process.

Use‐dependent learning is a simple and goal‐independent process that forms motor memories by changing movements to become more like the previous movement [8]. It has been consistently linked to changes in activity within M1 and S1 [9–11] and the premotor cortices. In contrast, reinforcement learning is the process whereby actions are chosen and repeated based on reward feedback, with successful actions being reinforced through reward maximization. It has been traditionally linked to activity in the basal ganglia (i.e., the “reward system”), although there is ample evidence that M1 also processes reward signals and plays an important role in reinforcement learning by integrating dopaminergic inputs and modulating plasticity in response to reward, thus actively connecting action execution and reward processing within cortical motor networks [12, 13]. Similarly, S1 also encodes rewards in mice and therefore may play a role in mediating reinforcement learning [14, 15].

Outputs generated in M1 are responsible for voluntary movements using the corticospinal tract [16]. M1 is organized as a relatively somatotopic map containing multiple motor synergies related to different functional movements, thus playing a crucial role in both storing and executing movement patterns and forming nodes that link motor and cognitive processes that are shaped by repetition and learning [17, 18]. In parallel, S1 has been traditionally considered as an area receiving somatosensory information from the periphery, organized similarly in relatively somatotopic maps. However, S1 also seems particularly important in ML. First, animal studies have demonstrated that damage to S1 hampers motor task learning [19]. Additionally, noninvasive brain stimulation applied to this area has been demonstrated to influence ML in healthy individuals [10]. Furthermore, a relationship has been observed between changes in short‐latency afferent inhibition and ML [20]. This type of inhibition is associated with somatosensory cortex excitability, and it is hypothesized that it reflects increased connectivity between S1 and M1 due to improved somatosensory information processing in S1 [20, 21]. However, the relative contribution of these two areas to different forms of learning remains uncertain.

Repetitive transcranial magnetic stimulation (rTMS) is a therapeutic tool currently used to enhance upper limb motor function after brain injury and to reduce the severity and frequency of chronic pain [22, 23]. It involves applying a magnetic current through two copper coils, generating an electric current in the brain, which can modulate local neuronal excitability in a region‐specific manner [24]. Thus, rTMS can be used to prove causal relationships between brain areas and behavioral phenomena. There are two primary rTMS approaches: high‐frequency rTMS (>5 Hz), which aims to increase neuronal excitability, and low‐frequency rTMS (1 Hz), intended to inhibit target neuron activity [25].

Although applying rTMS to M1 in individuals with neurological conditions has long been shown to be effective in improving motor recovery [23], results in healthy people are more variable. While physiological changes like increased corticospinal excitability are well documented, these do not always translate into better performance on tests that measure ML in healthy individuals [26–29]. The effects of rTMS applied to S1 in healthy individuals show similar results. A body of evidence supports its neurophysiological effects, which is significant considering S1’s role in ML. However, there is ongoing debate about its potential effects over ML in healthy people [10, 30–32]. This might be because M1 and S1 contribute differently to ML, promoting it via relatively different mechanisms and/or at different stages of skill acquisition.

Our study evaluates whether rTMS applied to M1 or S1 enhances ML, thereby highlighting their role in skill acquisition. Since previous rTMS studies targeting M1 in healthy adults have shown mixed results, we will compare stimulation sites using identical outcome tasks to eliminate task‐dependent confounds. We hypothesized that S1 stimulation would enhance use‐dependent learning more than M1 stimulation and sham and that M1 stimulation would enhance reinforcement‐based learning more than S1 stimulation and sham.

## 2. Materials and Methods

### 2.1. Study Design and Setting

A single‐blind randomized controlled trial (RCT) was carried out at the Neuron Clinic in Madrid, Spain, and reported following the CONSORT 2025 criteria [33]. This trial took place between January 2024 and July 2025.

### 2.2. Participants and Eligibility Criteria

The participants were healthy individuals recruited through snowball sampling. The inclusion criteria were as follows: (1) age between 18 and 65 years, (2) right‐hand dominance, and (3) no neurological or medical conditions. The exclusion criteria were as follows: (1) presence of any neurological disorder, (2) experiencing pain during the study period, (3) history of epileptic seizures, (4) metallic implants in the skull, (5) having electronic devices implanted, and (6) current treatment with antiepileptic or antidepressant medications. All participants provided written informed consent before participating in the study. The research adhered to the ethical standards for medical research involving human participants, as outlined in the Declaration of Helsinki. The study was approved by the “Regional Drug Research Ethics Committee of the Community of Madrid” (internal number: A003). We measured the laterality quotient from the Edinburgh Handedness Inventory [34] and assessed physical activity using the International Physical Activity Questionnaire (IPAQ) [35] as baseline variables.

### 2.3. Randomization

Randomization was conducted utilizing a computer‐generated random sequence table with a balanced three‐block design using randomizer.org [36]. An independent researcher created the randomization list, and a team member uninvolved in the assessment or intervention procedures was responsible for overseeing the randomization process and safeguarding the list. Participants were randomly allocated to one of the three groups using concealed, sealed envelopes, thereby ensuring allocation concealment.

### 2.4. Blinding

Different researchers performed the assessments and interventions. The evaluator was blinded to the participant’s allocation during the measurements and data recording. Participants were instructed not to disclose to the researcher conducting the measurements the details or feelings about their stimulation. Therefore, the evaluator did not know at any time which intervention each participant received during the assessment. However, the researcher who performed the treatment knew which treatment was assigned to each participant, as this was required to appropriately position the TMS coil at the targeted brain region.

### 2.5. General Procedure

**Figure 1:**
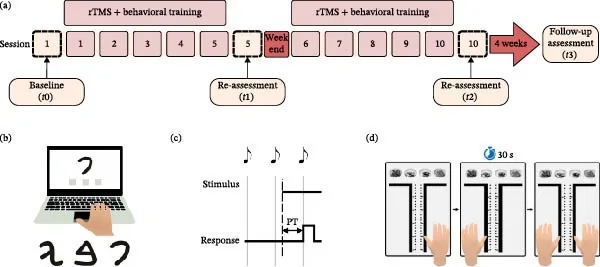
Experiment overview. (a) General procedure scheme. “Behavioral training” includes an arbitrary visuomotor association (AVMA) task and the Purdue Pegboard Test (PPT). (b) Experimental setup for the AVMA and the forced response‐AVMA (FR‐AVMA). Participants had to associate each symbol on the screen with a specific corresponding key (“J,” “K,” and “L”) using the index, middle, and ring fingers of the right hand. (c) To assess performance before and after training, a FR‐AVMA procedure was used. Participants were instructed to respond synchronously with the final tone in a sequence of three equally spaced tones. (d) Experimental setup for the PPT. Participants had to place as many pegs as possible in 30 s. The PPT was always performed first with his/her right hand, then with the left hand, and finally with both hands at the same time.

After randomization and signing informed consent, the researcher recorded the participant’s baseline variables and sociodemographic data. The study was conducted over 10 workdays, with 2 days’ rest during the weekend after the first 5 days, followed by a 4‐week follow‐up. On the first day, participants completed a baseline assessment with the two outcome measures (*t*0), and immediately afterwards, they received the first rTMS intervention according to their group and began behavioral training (see below Figure 1).

The training was performed daily, and reassessments were conducted on days 5 (*t*1) and 10 (*t*2) after the training sessions. Finally, a reassessment session (*t*3) was carried out 4 weeks after completing the treatment protocol as a retention test (Figure 1a).

### 2.6. Interventions

#### 2.6.1. Behavioral Training

All participants in each group received the same behavioral intervention that consisted of two different motor tasks that were always completed in the same order. This intervention was always practiced after the participants received their assigned rTMS protocol.

First, they completed a gamified task, the arbitrary visuomotor association (AVMA) task [37], which consisted of five blocks of 100 reaction‐time trials (500 trials/session). In each trial, an arbitrary visual stimulus (one of three letters from the Phoenician alphabet) appeared at the center of the screen, and participants were required to respond using the corresponding key (“J,” “K,” or “L” with their right index, middle, and ring fingers). Correct responses produced a pleasant tone, and after a 300 ms delay, the next trial began. Incorrect responses triggered a buzzer sound, followed by a mandatory 1000 ms delay, after which the same stimulus was presented again until the correct response was provided. At the end of each block, participants received feedback regarding their completion time and its comparison to their personal best of that session. They were instructed to improve their performance by attempting to surpass their previous best time in each subsequent block (Figure 1b).

Lastly, participants completed a second task—the Purdue Pegboard Test (PPT)—where they aimed to place as many pegs as possible within 30 s [38]. The task was performed in the following sequence: first with the right hand, then with the left hand, followed by both hands simultaneously, and this pattern was repeated until they completed four trials of each type (right hand, left hand, and both hands) in each training session (Figure 1d).

#### 2.6.2. rTMS Intervention

Two experimental groups and a sham group were designed to evaluate the effectiveness of rTMS on ML when applied over M1 and S1. Stimulation was delivered using the STM 9000, ATES MEDICA Device (EBNeuro SpA, Firenze, Italy), with a 70 mm air‐cooled, flat, figure‐of‐eight coil. The participants sat in a chair with back support in a relaxed posture throughout the TMS procedure.

For M1 stimulation, the target area was identified as the motor hotspot of the first dorsal interosseous (FDI) muscle of the dominant hand. The hotspot was identified by positioning the coil over the head area that produced the maximum response in FDI, placing the coil tangentially to the scalp ~3 cm lateral to the midline and the coil handle oriented with 45° rotation; however, the exact position was adjusted individually [39]. Then, the resting motor threshold (RMT) was determined individually, defined as the lowest intensity (expressed as % of maximal stimulator output) that produced a visible contraction of the FDI muscle in at least five of 10 consecutive trials [40]. This measurement was performed daily prior to stimulation. Repetitive stimulation was administered at 90% of the RMT. Each session consisted of 880 pulses delivered at 10 Hz in 22 trains of 4 s, with 10‐s intertrain intervals [27].

For S1 stimulation, the same protocol was used, with the coil placed 2 cm posterior to the M1 hotspot. To ensure correct positioning, 10 single pulses at 110% of the RMT were applied, confirming the absence of motor responses, as described in previous studies [41, 42].

Finally, participants in the control group received sham rTMS stimulation. Identification of the motor hotspot and RMT recording was conducted daily, just like in the intervention groups, but the coil was placed perpendicular to the skull, on the motor hotspot, during the repetitive stimulation protocol, which drastically reduces the electric field induced in the cortex and thus prevents neuronal activation [43]. This is a previously validated setup that reproduces the noise and vibration characteristic of stimulation but does not produce effective cortical stimulation, thus acting as a placebo condition.

### 2.7. Outcome Measures

The primary outcome measure was the average number of pegs placed on the PPT, calculated separately as the mean across three trials per hand condition (right, left, and both hands) at each assessment timepoint. Separate scores for the right hand, left hand, and both hands simultaneously (always acquired in this order) were computed [44]. The PPT predominantly taps into practice‑related, use‑dependent improvements in manual dexterity, although additional processes such as procedural learning and strategy use may also contribute [45].

As a secondary outcome measure, accuracy during a forced response (FR) version of the AVMA (FR‐AVMA) was used. As opposed to “free” reaction time paradigms, in FR paradigms, the time allowed to process the stimulus is systematically manipulated trial‐to‐trial, therefore allowing to include it as an independent variable (time used to prepare a response or preparation time). As such, FR paradigms measure accuracy as a function of response time, effectively capturing the speed‐accuracy trade‐off (SAT) for a task and involving reinforcement‐like learning while still incorporating other components such as error‐based correction and habit formation.

The protocol was similar to Hardwick et al. [46]. Before completing the main task, participants first learned the timing of their responses in a familiarization section, as this is a critical requirement in FR protocols. Relative to a series of three auditory tones, each separated by 400 ms, they were instructed to synchronize their response (pressing a randomly assigned key: “J,” “K,” or “L”) with the onset of the third tone (Figure 1c). The visual stimulus was presented at a randomly determined time (uniform distribution from 40 to 800 ms in steps of 10 ms) within the tone sequence, and participants received feedback on whether their response was “too early” or “too late,” defined as more than 100 ms before or after the third tone.

After four blocks of 15 practice trials, participants learned to associate specific visual stimuli—symbols representing letters from the Phoenician alphabet—with corresponding responses (keys “J,” “K,” or “L”), i.e., the AVMA. Each symbol‐key mapping was practiced until the participant achieved five consecutive correct responses per symbol.

In the FR‐AVMA procedure, participants completed four blocks of 100 trials each, using the same timing paradigm as in the familiarization section but now responding according to the symbol‐key associations. The stimulus was again presented at a randomly distributed time within the tone sequence. When the stimulus appeared very shortly (less than ~200 ms) before the third tone, participants had insufficient time to process it and were effectively forced to guess, yielding an expected accuracy of one‐third. Each trial therefore provided a measure of the participant’s accuracy (correct or incorrect) at a given preparation time (the time between the appearance of the stimulus and the participant’s response). Combining data across multiple trials therefore allowed us to measure a SAT for performance.

### 2.8. Sample Size Calculation

A sample size calculation was performed based on statistical precision to detect a difference in means between two groups for the PPT. No similar studies have been conducted that use noninvasive stimulation as the primary variable. The “ss.aipe.c.ancova” function from the MBESS package in R (version 4.5) was used [47]. Using the study by Pixa et al. [48] as reference, a standard deviation similar to that observed for the PPT was considered (experimental groups: 6 points; control group: 4 points). The correlation between the baseline and postintervention scores was set at *r* = 0.5, as no previous studies exist, but a moderate correlation is expected for repeated measures in the same subject within a period of less than 2 years [49]. The precision range for the difference in means was set at ±3 points, with an expected power (1‐beta) of 0.8, as the previous study had observed these differences between groups along with significant changes. The confidence interval was set at 0.95, and an expected attrition rate of 10% was included. According to these parameters, the total sample size needed for the study, accounting for participant loss, was 20 participants per group.

### 2.9. Data Analysis

All analyses were performed in R version 4.5.0 [50]. Scripts and data are freely available at zenodo.org (https://doi.org/10.5281/zenodo.17871901).

As a manipulation check, we confirmed that the behavioral training protocols showed an effect on both PPT’s number of pegs and reaction times and errors in the “free reaction time” AVMA task across groups. This only took into consideration the data collected during the training sessions and not during the formal assessments (*t*1, *t*2, and *t*3). For that, we ran generalized linear mixed models (GLMMs) whose details are provided in Supporting Information.

For the PPT, various GLMMs were compared with different link functions (including a linear model, a gamma model, and a beta‐regression model) and the baseline measure as the covariate (ANCOVA model). The model with the lowest Akaike’s Information Criterion was selected. This resulted in the beta‐regression model being the final choice. The model included the participant as a random intercept and considered the within‐subject effects of time (*t*1, *t*2, and *t*3), type of PPT (right, left, or both hands), and the between‐subject effect of group (S1, M1, or Sham) and their interaction by the formula: PPT ~ Baseline + Time ^∗^Type ^∗^Group + (1|Participant). Post hoc a priori analysis was performed comparing the groups pairwise for each level of time and type, obtaining as evidence for treatment effects the odds ratios, 95% confidence intervals (95%CIs), and Bonferroni‐corrected *p*‐values. Statistically significant differences were considered when *p*  < 0.05.

In the analysis of the FR‐AVMA task, we first excluded participants who provided less than 50% of their responses in the prespecified valid time window (+/−100 ms from last tone), therefore not adhering to the timing instruction. This resulted in excluding two participants from *t*1, one participant from *t*2, and zero participants from *t*3. The data were trimmed to exclude trials where implausible preparation times were recorded (e.g., negative times, suggesting the response was provided before the stimulus appeared or preparation times >1000 ms, indicative of a technical error) or no response was provided. The final datasets of the postintervention assessments were analyzed using generalized additive mixed models (GAMMs) as they allow modeling of nonlinear relationships between predictors and response via smooth functions [51]. A separate model was built for each session, each with the same structure: trial‐level accuracy (outcome) was modeled with a binomial distribution (0–1 binary: correct/incorrect), with predictors: preparation time as a continuous smooth covariate and its interaction with treatment allocation, coded as a categorical predictor (S1, M1, and sham groups). Models were built with parameter *k* = 20 (number of smooth bases), as previous work indicates that this provides enough flexibility to capture data from similar FR procedures [52]. Thin‐plate regression splines were used, with 2nd derivative penalization to control overfitting, using restricted maximum likelihood estimation [53]. The participants were included as a random effect (random intercept). Goodness‐of‐fit was assessed via the *k*‐index (values > 0.95 were considered appropriate) and comparing the effective degrees of freedom (edf) to the allowed degrees of freedom [54]. Model‐derived predictions were computed and illustrated via SATs, with the probability of a correct response (proportion correct) plotted against preparation time and uncertainty as 95%CIs derived from 1000 bootstrapped replicates. Differences in probabilities between the groups were computed and statistically compared via *z*‐tests, correcting for multiple comparisons by the false discovery rate (FDR) procedure.

## 3. Results

**Figure 2:**
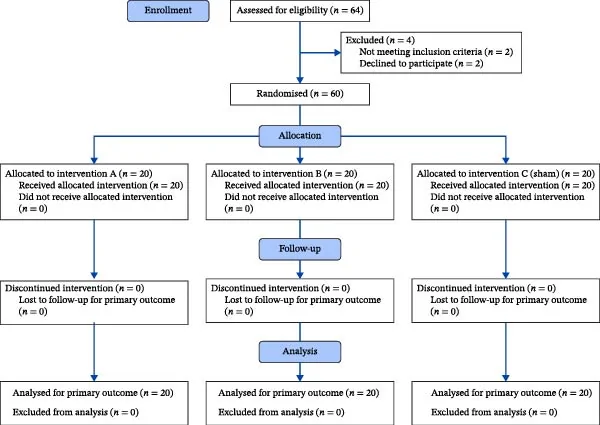
Flowchart diagram of the participants in the study.

A total of 60 participants were included and randomly assigned to three groups of 20 participants each. All participants received the interventions as intended and attended the assessment sessions, without dropouts or missing data (Figure 2). No adverse events were reported in any group. Table 1 shows the baseline variables for each group.

**Table 1:** Participant characteristics at baseline.

Characteristic	M1 (*N* = 20)	S1 (*N* = 20)	Sham (*N* = 20)	Overall (*N* = 60)
Age^a^	28.15 (10.76)	27.00 (8.99)	32.50 (12.15)	29.22 (10.80)
Sex^b^				
Female	10 (50%)	13 (65%)	10 (50%)	33 (55%)
Male	10 (50%)	7 (35%)	10 (50%)	27 (45%)
IPAQ^a^	4659.25 (4273.99)	3920.43 (2825.16)	3186.58 (2154.51)	3922.08 (3211.75)
PPT (right hand)^c^	14.73 (1.31)	15.71 (1.50)	15.25 (1.19)	15.23 (1.38)
PPT (left hand)^c^	13.50 (1.80)	14.15 (1.50)	14.23 (1.52)	13.96 (1.62)
PPT (both hands)^c^	11.13 (1.20)	11.63 (1.50)	11.47 (1.19)	11.41 (1.30)

Abbreviations: IPAQ, International Physical Activity Questionnaire; PPT, Purdue Pegboard Test.

^a^Mean (SD).

^b^ *n* (%).

^c^Number of pegs inserted in 30 s (average of 3 attempts).

### 3.1. Manipulation Check

For the PPT training analysis, the effect of training session showed a significant increase in the number of pegs for the right hand (slope = 0.28 [0.25, 0.31], *t*_(7178)_ = 18.82, *p* = 3.41 × 10^−77^; Figure S1), left hand (slope = 0.28 [0.25, 0.31], *t*_(7178)_ = 18.82, *p* = 3.63 × 10^−77^; Figure S1), and both hands (slope = 0.25 [0.22, 0.28], *t*_(7178)_ = 16.38, *p* = 3.03 × 10^−59^; Figure S1). For the “free reaction time” AVMA, the effect of training session showed a significant reduction in reaction time (slope = −7.86 ms [−8.85, −6.87], *t*_(309462)_ = −15.5, *p* = 3.53 × 10^−54^; Figure S2) and the probability of an error (for each block of 100 trials; slope = ‐0.14% [−0.08, −0.2], *z* = 4.57, *p* = 4.9 × 10^−6^; Figure S2).

### 3.2. PPT

**Figure 3:**
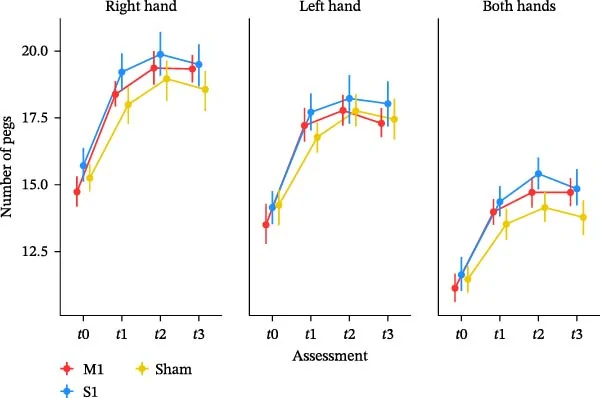
Group comparison for PPT performance by timepoint and hand.

Table 2 and Figure 3 reflect detailed results for PPT, which showed effect sizes ranging from small to moderate. The predefined post hoc analysis revealed several effects in favor of S1 over sham stimulation, with significant differences between the groups for the right hand at *t*1 (OR = 1.77, 95%CI [1.05–2.96], *p* = 0.025), both hands at *t*2 (OR = 1.96, 95%CI [1.17–3.29], *p* = 0.006), and both hands at *t*3 (OR = 1.71, 95%CI [1.02 – 2.87], *p* = 0.039). The rest of the comparisons revealed no significant differences between groups (OR = 0.68–1.66, *p*  > 0.05).

**Table 2:** Pairwise group contrasts for PPT performance by timepoint and hand.

Time	Type	Contrast	Odds ratio (SE)	95%CI	*Z*	*p*‐Value
*t*1	Right	S1/M1	0.73 (0.16)	[1.23–0.43]	−1.44	0.449
		M1/sham	1.29 (0.28)	[2.16–0.77]	1.19	0.698
		S1/sham	1.77 (0.38)	[2.96–1.05]	2.64	**0.025**
	Left	S1/M1	0.80 (0.17)	[1.35–0.48]	−1.01	0.931
		M1/sham	1.33 (0.29)	[2.23–0.79]	1.31	0.573
		S1/sham	1.66 (0.36)	[2.79–0.98]	2.32	0.061
	Both	S1/M1	0.82 (0.18)	[1.38–0.49]	−0.90	1.000
		M1/sham	1.28 (0.27)	[2.13–0.76]	1.14	0.763
		S1/sham	1.55 (0.34)	[2.60–0.93]	2.03	0.126
*t*2	Right	S1/M1	0.79 (0.17)	[1.33–0.47]	−1.06	0.867
		M1/sham	1.26 (0.27)	[2.11–0.76]	1.09	0.831
		S1/sham	1.59 (0.34)	[2.66–0.95]	2.15	0.095
	Left	S1/M1	0.77 (0.17)	[1.30–0.46]	−1.17	0.721
		M1/sham	1.14 (0.25)	[1.92–0.68]	0.60	1.000
		S1/sham	1.47 (0.32)	[2.47–0.87]	1.78	0.226
	Both	S1/M1	0.68 (0.15)	[1.13–0.40]	−1.82	0.208
		M1/sham	1.32 (0.28)	[2.21–0.79]	1.31	0.570
		S1/sham	1.96 (0.42)	[3.29–1.17]	3.12	**0.005**
*t*3	Right	S1/M1	0.92 (0.20)	[1.56–0.55]	−0.36	1.000
		M1/sham	1.48 (0.32)	[2.47–0.88]	1.81	0.209
		S1/sham	1.60 (0.34)	[2.68–0.95]	2.16	0.091
	Left	S1/M1	0.74 (0.16)	[1.25–0.44]	−1.38	0.501
		M1/sham	1.05 (0.23)	[1.76–0.62]	0.22	1.000
		S1/sham	1.42 (0.31)	[2.38–0.84]	1.60	0.326
	Both	S1/M1	0.91 (0.20)	[1.52–0.54]	−0.45	1.000
		M1/sham	1.55 (0.33)	[2.60–0.93]	2.05	0.120
		S1/sham	1.71 (0.37)	[2.87–1.02]	2.49	**0.038**

*Note:* Bold values indicate statiatical significance at *p*<0.05.

### 3.3. FR AVMA (FR‐AVMA) Task

**Figure 4:**
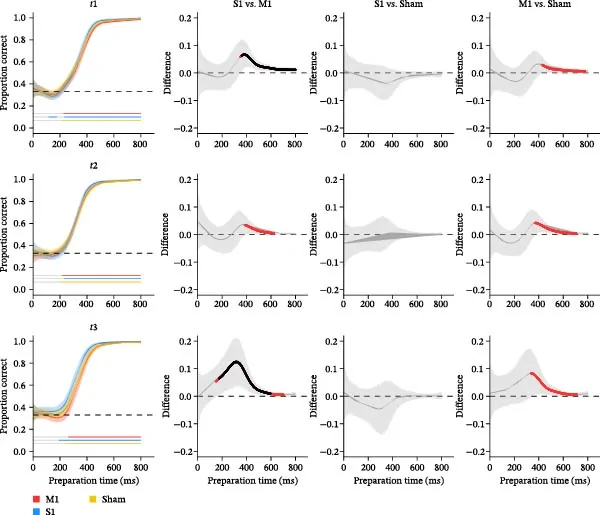
FR‐AVMA accuracy across preparation time by group and session, with model‐based pairwise contrasts.

In the AVMA task, 97.46% ± 1.77, 96.84% ± 2.38%, and 97.49% ± 1.87 trials were available for the FR analysis at *t*1, *t*2, and *t*3, respectively. We first confirmed nonlinearity of preparation time in each GAMM (*t*1: edf = 9.23, *p*  < 2 × 10^−16^; *t*2: edf = 7.36, *p*  < 2 × 10^−16^; *t*3: edf = 9.0; *p*  < 2 × 10^−16^). Overall, all groups showed a proportion of correct responses above chance level (33%) at around ~200–240 ms and reached 95% accuracy at around ~450–500 ms (Figure 4).

No differences between groups were found at *t*0 (Figure S3). At *t*1, differences in favor of S1 stimulation were found between S1 and M1 groups in the period 360–800 ms (peak = −0.07 [−0.11, −0.02], *z* = −2.66, and *p*_FDR_ = 0.017; Figure 4, upper panel). No differences between S1 and sham (peak = −0.04 [−0.10, 0.02], *z* = −1.29, *p*_FDR_ = 0.522; Figure 4, upper panel) or between M1 and sham (peak = 0.03 [−0.01, 0.073], *z* = 1.6, *p*_FDR_ = 0.217; Figure 4, upper panel) were found. At *t*2, no differences were found between any of the groups (differences < 0.05, *z* < 2.02, *p*  > 0.107; Figure 4, middle panel). Finally, at *t*3, again, differences in favor of S1 stimulation were found when comparing between S1 and M1 in the period 176–598 ms (peak = −0.12 [−0.21, −0.04], *z* = −2.87, *p*_FDR_ = 0.029; Figure 4, lower panel) but not between S1 and sham (peak = −0.05 [−0.14, 0.04], *z* = −1.05, *p*_FDR_ = 0.999; Figure 4, lower panel) or M1 and sham (peak = 0.08 [0.00, 0.16], *z* = 1.96, *p*_FDR_ = 0.106; Figure 4, lower panel).

## 4. Discussion

This study aimed to compare different brain stimulation sites (S1 and M1) to ascertain the role of these brain regions in different aspects of ML in healthy individuals. The executive, use‐dependent process was assessed through the PPT (focused on dexterity), which we primarily interpret as reflecting use‐dependent improvements, even though other forms of learning undoubtedly contribute to performance. We found limited evidence of improvement after S1 stimulation compared to sham but no differences between S1 and M1 stimulation or M1 stimulation against sham. The FR‑AVMA procedure was used to examine reinforcement‑like learning under time pressure and reward feedback while recognizing that it also involves broader procedural and error‑based learning mechanisms. These FR‐AVMA analyses were exploratory and may have been underpowered relative to the primary endpoint. We did not find convincing evidence that either rTMS protocol was able to generate significant effects compared to the sham.

### 4.1. PPT

PPT is usually used for measuring dexterity, which is a more executive aspect of ML [55]. Certain studies have identified a correlation between dexterity, as measured with PPT, and the ability to acquire skills [56, 57]. For example, Sun et al. [57] found differences between musicians and nonmusicians in PPT scores and in various surgical tasks that participants were required to learn. In our study, we identified certain effects of S1 stimulation; we observed an early improvement at *t*1 only in the right (dominant) hand for the S1 group, which corresponds to the stimulated (left) hemisphere. However, no significant differences were found between groups at *t*2 or *t*3, likely due to a ceiling effect in the test. This phenomenon has also been reported in previous studies, where performance tended to plateau after approximately 2 weeks of training [58]. This aligns with our findings and may explain why only the most complex PPT subtest, which requires the coordinated use of both hands and imposes higher motor demands, continued to show between‐group differences, as the performance ceiling was not yet reached for this task.

However, considering the likelihood of the relatively small absolute changes observed, these results should be interpreted as indicating modest performance advantages rather than a strong, generalized improvement of dexterity‐related ML in healthy individuals.

Although there is indirect evidence that suggests improvements in ML after applying rTMS over S1 in healthy participants [10, 59, 60], few studies have examined the effectiveness of enhancing the neural excitability of this region through any form of brain stimulation. Similar to our approach, Platz et al. [30] investigated the effectiveness of intermittent theta‐burst stimulation applied over S1, M1, and a sham condition on ML tasks after 5 days of training. Their results showed statistically significant differences between the experimental and sham groups but not between the two experimental conditions. However, interpreting these findings in relation to our results is challenging because the authors did not present the outcomes separately for each stimulation site (M1 and S1), which limits our ability to determine whether S1 stimulation produced effects comparable to those observed in our study. Moreover, the outcome measures used in that study, the arm ability task and the nine‐hole peg test, are validated tools for assessing upper limb function in clinical populations. Nonetheless, both tests are prone to ceiling effects, particularly in participants with mild impairment or after repeated practice. This methodological aspect might have constrained the sensitivity to detect further improvements, potentially contributing to the small effect size reported (~2% improvement in the arm ability task).

Similar to us, Choi et al. [61] applied excitatory rTMS over S1 and found improvements in ML using a paradigm based on synchronizing the activation of two muscles at the same time. Furthermore, Brodie et al. [62] applied rTMS over S1 in stroke patients, observing differences in favor to the experimental group compared with sham stimulation in a ML task based on a serial tracking paradigm but not in motor function. However, their study did not assess changes in activity, which could be more similar to our dexterity paradigm. On the other hand, several studies have reported that high‐frequency rTMS over M1 increases corticospinal excitability and shortens reaction times [28, 63–67]. However, the study carried out by Weiler et al. [68], which assessed ML using dexterity‐based outcomes, did not find statistically significant differences compared with the sham group after M1 stimulation, which aligns with our findings. Importantly, their protocol differed from ours because they applied low‐frequency rTMS to the behaviorally trained M1. Opposite to these results, the study by Platz et al. [30] found statistically significant differences in the arm ability task for both M1 and S1 stimulations compared with sham. However, as previously mentioned, it is difficult to determine the effects of each type of stimulation. In contrast to us, Pixa et al. [48] observed significant differences between the experimental and control groups after 3 days of anodal transcranial direct‐current stimulation. Given that this type of electrical stimulation is less spatially selective than rTMS, these improvements may reflect nonspecific modulation beyond M1 due to more diffuse stimulation of adjacent, functionally related regions such as premotor cortex or S1 [69–72].

Our research suggests positive effects of rTMS applied over S1 in favoring ML during a dexterity task at intervals of 5 days (*t*1, right hand), 10 days (*t*2, both hands), and 4 weeks subsequent to treatment (*t*3, both hands). Dexterity, or skill acquisition, comprehends three stages, starting with cognition (understanding the task), followed by integration (motor skills are applied to increase efficiency), and automation (no longer needs external cues) [73]. In this context, plastic changes in S1 have been shown to predict ML in this type of tasks and disrupting them with continuous theta‐burst stimulation blocks motor memory consolidation [59, 60]. Indeed, S1 seems to play a key role in consolidating newly learned movements, which is consistent with the improvements observed in our study for the PPT [74]. Considering this, our results suggest that the behavioral impact of excitatory S1 stimulation under the current protocol is limited in scope and should be interpreted as representing minor changes in performance rather than a robust enhancement of ML. In fact, S1 probably functions as part of a larger, distributed network, and future studies with larger sample sizes and outcome measures less affected by ceiling effects will be necessary to better understand the extent and reliability of these effects.

Although current evidence supports that cognitive and motor processes operate through a distributed network of interconnected brain regions [18], M1 does not appear to be the main driver of ML according to our results. The present findings do not suggest that M1 is unrelated to ML, and its involvement is well established in the literature [11, 75], but rather that excitatory stimulation of M1 does not necessarily enhance ML outcomes in healthy individuals. M1 is highly interconnected with regions such as the cerebellum and S1, both crucial for ML [76, 77]; however, being part of this network does not imply having a dominant causal influence on learning. In line with this, Psurek et al. [78] applied inhibitory rTMS over M1, similar to Vidoni et al. [10], who did so over S1, and found that inhibition of M1 did not impair ML, further supporting the idea that modulating M1 alone may be insufficient to alter the learning process.

### 4.2. FR‐AVMA

This is the first study to assess the effects of rTMS using the FR‐AVMA, which limits the ability to compare its results to others in the literature. Our training paradigm was similar to previous studies [37] and can be interpreted in the context of habit formation and reinforcement learning. These physiological processes rely on partially overlapping neural systems that integrate sensory, motor, and reward‐related information to optimize behavior. While the basal ganglia, particularly the dorsal striatum, have been crucially linked to this process by encoding stimulus–response associations, dopaminergic projections from the midbrain convey reward prediction error signals that reinforce successful actions and guide plasticity within cortico‐striatal loops [8, 79]. As behavior transitions from goal‐directed to habitual, activity shifts from ventromedial prefrontal and dorsomedial striatal regions toward SM cortical areas and the dorsolateral striatum, reflecting a redistribution of control from deliberate to automatic processes [8].

Recent evidence suggests that M1 and S1 cortices are not merely passive executors of movement but active components in reinforcement‐driven plasticity and habit consolidation [9, 10, 75]. Studies in animals and humans have shown that M1 encodes both reward‐related and error‐based signals, integrating dopaminergic inputs from midbrain structures such as the ventral tegmental area to reinforce successful motor outputs [75, 80]. This dopaminergic modulation promotes long‐term potentiation of specific motor representations, strengthening stimulus–response associations that underlie habitual performance [81–83]. Similarly, S1 contributes to SM prediction and feedback evaluation, providing precise sensory error signals that interact with reward‐based reinforcement to refine motor commands [84]. Over repeated practice, these cortices may act as local hubs of experience‐dependent plasticity, where the convergence of sensory and reward information facilitates the automatization of motor behaviors and the stabilization of learned habits [85]. These neural dynamics provide a mechanistic framework for interpreting ML paradigms such as FR‐AVMA within the broader context of reinforcement‐driven plasticity.

However, based on our findings, it cannot be inferred that high‐frequency rTMS over M1 or S1 influences ML as assessed through the FR‐AVMA paradigm in healthy individuals. Although our results show a tendency for S1 stimulation to enhance ML and even a slowdown with M1 stimulation (in particular at t3, the retention measure), no statistically significant differences were found between any active group and the control group. This null result may reflect a genuine lack of effect, insufficient statistical power for an effect size smaller than expected, or limitations in the sensitivity of the FR‐AVMA measure. However, because S1 stimulation tended to improve performance and M1 stimulation did not show this effect, we did find differences between S1 and M1 stimulation. Previous studies employing similar behavioral paradigms such as the serial reaction time task to assess ML have reported mixed results [64–67, 86]. Some studies show statistically significant improvements in the stimulation group, including fewer errors and faster reaction times in the serial task [65, 66, 86], while others report no differences between the groups [64, 67]. The discrepancies in the literature may be attributed to several factors, including small sample sizes (typically fewer than 20 participants per group), short durations of the behavioral interventions, and variations in the protocols employed. Notably, no studies have assessed the effectiveness of rTMS applied over M1 for more than 2 days. While Wessel et al. [64] and Rumpf et al. [67] employed three or 10 stimulations, respectively, they applied all stimulations on the same day.

The effectiveness of rTMS relies on plasticity‐driven changes that are enhanced over consecutive days of stimulation [87–89]. Therefore, our study is the first to explore the effectiveness of rTMS applied over multiple days on the formation of habits/reinforcement learning. Our results showed no differences between the active groups and the sham group, suggesting that rTMS over S1 or M1 may not be effective in improving reinforcement ML as assessed through the FR paradigm, although both groups showed opposite tendencies (in particular at retention). This may be explained by the fact that the FR protocol in the present study provided was not precise enough for a between‐group comparison with our sample sizes. As our protocol was already fatiguing for the participants due to the inclusion of several tasks, we decided to collect the minimum number of trials required to obtain reliable measures. However, the high variability in performance across trials, due to the complexity of the task, may have made the interpretation of the results challenging. Thus, increasing the number of trials and/or participants could strengthen the robustness of our findings. Furthermore, all three groups attained nearly ceiling levels of accuracy (~95%–97%) at preparation times exceeding roughly 450–500 ms. This significantly narrows the range within which the detection of stimulation‐related enhancements is possible. Differences between groups are most likely to manifest within the transition zone (~200–400 ms), where performance escalates sharply from the chance level to nearly perfect accuracy.

## 5. Strengths and Limitations

This study has several limitations that should be considered when interpreting the findings. First, the sample size was based on the PPT and may therefore have been underpowered to detect modest between‐group effects in the AVMA, especially after multiple‐comparison adjustments and when modeling effects across the preparation time. Furthermore, the signal‐to‐noise ratio of the FR‐AVMA task used here may have been limited. Between‐group contrasts tend to concentrate within relatively narrow temporal windows, which reduces effective power. Increasing the number of trials per session, using adaptive sampling around the transition region (~200–500 ms), and/or estimating individual psychometric parameters could improve the signal‐to‐noise ratio and the stability of estimates.

On the other hand, the rTMS intervention duration (10 sessions) may have been insufficient to induce robust and sustained neural changes that could be detected behaviorally, particularly for processes that rely on consolidation or habit formation. It is plausible that longer or higher‐dose protocols (more sessions or booster sessions during follow‐up) would yield larger effects; therefore, the present results should be viewed as conservative.

Regarding accurate localization, we relied on the 2 cm rule to stimulate over S1, as we did not have access to neuronavigated localizers. This rule, as other similar ones like Beam‐F3 to localize the DLPFC, assumes similar head dimensions across individuals and has been challenged [90]. Furthermore, the sham condition likely reproduced some sound and sensory experiences, but it may not have fully matched the perceptual effects of active stimulation. Therefore, some limitation in blinding cannot be excluded.

Additionally, the study was conducted in healthy participants, which limits clinical generalizability; potential ceiling/floor effects in some measures (e.g., PPT) may influence the detection of subtle changes. Finally, we did not include any neurophysiological measurements, which could have shed light on the subtle changes observed behaviorally.

Finally, a further limitation concerns the mapping between behavioral tasks and specific learning mechanisms. In line with previous work [56, 57], we treated PPT performance as a proxy for use‑dependent learning and FR‑AVMA performance as a proxy for reinforcement‑like learning; however, neither task isolates a single process. Both likely involve overlapping contributions from procedural learning, error‑based learning, and SM adaptation, among others, and our inferences about “use‑dependent” and “reinforcement‑like” learning should therefore be regarded as approximate, task‑based interpretations rather than process‑pure measures.

## 6. Conclusions

High‐frequency rTMS over S1 produced selective, time‐limited improvements in manual dexterity, whereas M1 stimulation did not differ significantly from either S1 or sham. These findings suggest that S1 may contribute to use‐dependent learning under excitatory rTMS, although the effects were modest and limited to selected measures. On a task assessing reinforcement‐like learning, neither rTMS protocol differed significantly from sham; however, S1 and M1 displayed opposite numerical tendencies at retention, with S1 trending toward improved and M1 toward reduced accuracy, yielding a significant S1–M1 contrast. Future studies should examine dose–response relationships, neural mechanisms via neuroimaging, and potential translation to clinical populations with motor impairments.

## Funding

The project has been partially funded through a competitive call for proposals, via the 5th Call for Research Grants awarded by the Professional Association of Physiotherapists of the Community of Madrid. Marcos Moreno‐Verdú is supported by an FNRS ‘Chargé de Recherches’ (CR) postdoctoral fellowship (Grant FNRS 1.B359.25), and Robert Hardwick is supported by an FNRS ‘MIS’ grant (Grant FNRS F.4523.23).

## Conflicts of Interest

The authors declare no conflicts of interest.

## Supporting Information

Additional supporting information can be found online in the Supporting Information section.

## Supporting information


**Supporting Information** The Supporting Materials file includes additional methodological details and supporting results related to the manipulation checks conducted in this study. Figure S1: presents the results of the training analysis for the Purdue Pegboard Test (PPT) across all training sessions. Results show that all three outcome measures (left hand, right hand, and both hands) improved progressively with training, regardless of the rTMS protocol received. Figure S2: presents the results of the training analysis for the rapid response arbitrary visuomotor association (AVMA) task. Panel a displays reaction time, and panel b displays the number of errors across training sessions, both showing improvement over time. Figure S3: presents the results of the forced response arbitrary visuomotor association (FR‐AVMA) task at baseline (*t*0). Panel a shows the model‐based speed‐accuracy trade‐off function for each group, while panels b–d display between‐group differences in the proportion of correct responses. No statistically significant between‐group differences were found at baseline after correcting for multiple comparisons using the false discovery rate, confirming group equivalence prior to the intervention.

## Data Availability

The anonymized dataset, analysis code, and materials are publicly available at zenodo.org (doi: 10.5281/zenodo.17871901).

## References

[1] Schmidt R. A. and Lee T. D. , Motor Control and Learning: A Behavioral Emphasis, 2011, 5th edition, Human Kinetics.

[2] Hikosaka O. , Nakamura K. , Sakai K. , and Nakahara H. , Central Mechanisms of Motor Skill Learning, Current Opinion in Neurobiology. (2002) 12, no. 2, 217–222, 10.1016/S0959-4388(02)00307-0. 12015240

[3] Lehéricy S. , Benali H. , and Van De Moortele P.-F. , et al.Distinct Basal Ganglia Territories are Engaged in Early and Advanced Motor Sequence Learning, Proceedings of the National Academy of Sciences. (2005) 102, no. 35, 12566–12571, 10.1073/pnas.0502762102. PMC1194910 16107540

[4] Coynel D. , Marrelec G. , and Perlbarg V. , et al.Dynamics of Motor-Related Functional Integration During Motor Sequence Learning, NeuroImage. (2010) 49, no. 1, 759–766, 10.1016/j.neuroimage.2009.08.048. 19716894 PMC2764831

[5] Lohse K. R. , Wadden K. , Boyd L. A. , and Hodges N. J. , Motor Skill Acquisition Across Short and Long Time Scales: A Meta-Analysis of Neuroimaging Data, Neuropsychologia. (2014) 59, 130–141, 10.1016/j.neuropsychologia.2014.05.001. 24831923

[6] Hardwick R. M. , Rottschy C. , Miall R. C. , and Eickhoff S. B. , A Quantitative Meta-Analysis and Review of Motor Learning in the Human Brain, NeuroImage. (2013) 67, 283–297, 10.1016/j.neuroimage.2012.11.020. 23194819 PMC3555187

[7] Leech K. A. , Roemmich R. T. , Gordon J. , Reisman D. S. , and Cherry-Allen K. M. , Updates in Motor Learning: Implications for Physical Therapist Practice and Education, Physical Therapy. (2022) 102, no. 1, 10.1093/ptj/pzab250, pzab250. 34718787 PMC8793168

[8] Spampinato D. and Celnik P. , Multiple Motor Learning Processes in Humans: Defining Their Neurophysiological Bases, The Neuroscientist. (2021) 27, no. 3, 246–267, 10.1177/1073858420939552. 32713291 PMC8151555

[9] Waiblinger C. , McDonnell M. E. , Reedy A. R. , Borden P. Y. , and Stanley G. B. , Emerging Experience-Dependent Dynamics in Primary Somatosensory Cortex Reflect Behavioral Adaptation, Nature Communications. (2022) 13, no. 1, 10.1038/s41467-022-28193-z, 534. PMC8795122 35087056

[10] Vidoni E. D. , Acerra N. E. , Dao E. , Meehan S. K. , and Boyd L. A. , Role of the Primary Somatosensory Cortex in Motor Learning: An rTMS Study, Neurobiology of Learning and Memory. (2010) 93, no. 4, 532–539, 10.1016/j.nlm.2010.01.011. 20132902

[11] Cirillo J. , Semmler J. G. , Mooney R. A. , and Byblow W. D. , Primary Motor Cortex Function and Motor Skill Acquisition: Insights From Threshold-Hunting TMS, Experimental Brain Research. (2020) 238, no. 7-8, 1745–1757, 10.1007/s00221-020-05791-1. 32222776

[12] Spampinato D. A. , Satar Z. , and Rothwell J. C. , Combining Reward and M1 Transcranial Direct Current Stimulation Enhances the Retention of Newly Learnt Sensorimotor Mappings, Brain Stimulation. (2019) 12, no. 5, 1205–1212, 10.1016/j.brs.2019.05.015. 31133478 PMC6709642

[13] Thabit M. N. , Nakatsuka M. , Koganemaru S. , Fawi G. , Fukuyama H. , and Mima T. , Momentary Reward Induce Changes in Excitability of Primary Motor Cortex, Clinical Neurophysiology. (2011) 122, no. 9, 1764–1770, 10.1016/j.clinph.2011.02.021. 21439903

[14] Lacefield C. O. , Pnevmatikakis E. A. , Paninski L. , and Bruno R. M. , Reinforcement Learning Recruits Somata and Apical Dendrites Across Layers of Primary Sensory Cortex, Cell Reports. (2019) 26, no. 8, 2000–2008.e2, 10.1016/j.celrep.2019.01.093. 30784583 PMC7001879

[15] Rabinovich R. J. , Kato D. D. , and Bruno R. M. , Learning Enhances Encoding of Time and Temporal Surprise in Mouse Primary Sensory Cortex, Nature Communications. (2022) 13, no. 1, 10.1038/s41467-022-33141-y, 5504. PMC9489862 36127340

[16] Graziano M. S. A. , Taylor C. S. R. , and Moore T. , Complex Movements Evoked by Microstimulation of Precentral Cortex, Neuron. (2002) 34, no. 5, 841–851, 10.1016/S0896-6273(02)00698-0. 12062029

[17] Graziano M. S. A. and Aflalo T. N. , Mapping Behavioral Repertoire onto the Cortex, Neuron. (2007) 56, no. 2, 239–251, 10.1016/j.neuron.2007.09.013. 17964243

[18] Gordon E. M. , Chauvin R. J. , and Van A. N. , et al.A Somato-Cognitive Action Network Alternates With Effector Regions in Motor Cortex, Nature. (2023) 617, no. 7960, 351–359, 10.1038/s41586-023-05964-2. 37076628 PMC10172144

[19] Pavlides C. , Miyashita E. , and Asanuma H. , Projection From the Sensory to the Motor Cortex is Important in Learning Motor Skills in the Monkey, Journal of Neurophysiology. (1993) 70, no. 2, 733–741, 10.1152/jn.1993.70.2.733. 8410169

[20] Mirdamadi J. L. and Block H. J. , Somatosensory Changes Associated With Motor Skill Learning, Journal of Neurophysiology. (2020) 123, no. 3, 1052–1062, 10.1152/jn.00497.2019. 31995429

[21] Turco C. V. , El-Sayes J. , Savoie M. J. , Fassett H. J. , Locke M. B. , and Nelson A. J. , Short- and Long-Latency Afferent Inhibition; Uses, Mechanisms and Influencing Factors, Brain Stimulation. (2018) 11, no. 1, 59–74, 10.1016/j.brs.2017.09.009. 28964754

[22] Beaulieu L.-D. and Milot M.-H. , Changes in Transcranial Magnetic Stimulation Outcome Measures in Response to Upper-Limb Physical Training in Stroke: A Systematic Review of Randomized Controlled Trials, Annals of Physical and Rehabilitation Medicine. (2018) 61, no. 4, 224–234, 10.1016/j.rehab.2017.04.003. 28579362

[23] Lefaucheur J.-P. , Aleman A. , and Baeken C. , et al.Evidence-Based Guidelines on the Therapeutic use of Repetitive Transcranial Magnetic Stimulation (rTMS): An Update (2014–2018), Clinical Neurophysiology. (2020) 131, no. 2, 474–528, 10.1016/j.clinph.2019.11.002. 31901449

[24] Abo M. and Kakuda W. , Rehabilitation With rTMS, 2015, 1st edition, Springer International Publishing.

[25] Klomjai W. , Katz R. , and Lackmy-Vallée A. , Basic Principles of Transcranial Magnetic Stimulation (TMS) and Repetitive TMS (rTMS), Annals of Physical and Rehabilitation Medicine. (2015) 58, no. 4, 208–213, 10.1016/j.rehab.2015.05.005. 26319963

[26] Bashir S. , Edwards D. , and Pascual-Leone A. , Neuronavigation Increases the Physiologic and Behavioral Effects of Low-Frequency rTMS of Primary Motor Cortex in Healthy Subjects, Brain Topography. (2011) 24, no. 1, 54–64, 10.1007/s10548-010-0165-7. 21076861 PMC3589808

[27] Di Lorenzo C. , Tavernese E. , and Lepre C. , et al.Influence of rTMS Over the Left Primary Motor Cortex on Initiation and Performance of a Simple Movement Executed With the Contralateral Arm in Healthy Volunteers, Experimental Brain Research. (2013) 224, no. 3, 383–392, 10.1007/s00221-012-3318-y. 23138522

[28] Yin Z. , Shen Y. , and Reinhardt J. , et al.5 Hz Repetitive Transcranial Magnetic Stimulation With Maximum Voluntary Muscle Contraction Facilitates Cerebral Cortex Excitability of Normal Subjects, CNS & Neurological Disorders - Drug Targets. (2015) 14, no. 10, 1298–1303, 10.2174/1871527315666151111124216. 26556078

[29] Wang X. , Li L. , and Wei W. , et al.Altered Activation in Sensorimotor Network After Applying rTMS Over the Primary Motor Cortex at Different Frequencies, Brain and Behavior. (2020) 10, no. 7, 10.1002/brb3.1670. PMC7375128 32506744

[30] Platz T. , Adler-Wiebe M. , Roschka S. , and Lotze M. , Enhancement of Motor Learning by Focal Intermittent Theta Burst Stimulation (iTBS) of Either the Primary Motor (M1) or Somatosensory Area (S1) in Healthy Human Subjects, Restorative Neurology and Neuroscience. (2018) 36, no. 1, 117–130, 10.3233/RNN-170774. 29439364

[31] Lerma-Lara S. , De Cherade Montbron M. , Guérin M. , Cuenca-Martínez F. , and La Touche R. , Transcranial Direct-Current Stimulation (tDCS) in the Primary Motor Cortex and Its Effects on Sensorimotor Function: A Quasi-Experimental Single-Blind Sham-Controlled Trial, Scientific Reports. (2021) 11, no. 1, 10.1038/s41598-021-85989-7, 6566. 33753853 PMC7985198

[32] Flix-Díez L. , Delicado-Miralles M. , Gurdiel-Álvarez F. , Velasco E. , Galán-Calle M. , and Lerma Lara S. , Reversed Polarity Bi-tDCS over M1 During a 5 Days Motor Task Training Did Not Influence Motor Learning. A Triple-Blind Clinical Trial, Brain Sciences. (2021) 11, no. 6, 10.3390/brainsci11060691, 691. 34070256 PMC8225177

[33] Hopewell S. , Chan A.-W. , and Collins G. S. , et al.CONSORT 2025 Statement: Updated Guideline for Reporting Randomized Trials, Nature Medicine. (2025) 31, no. 6, 1776–1783, 10.1038/s41591-025-03635-5. 40229553

[34] Albayay J. , Villarroel-Gruner P. , Bascour-Sandoval C. , Parma V. , and Gálvez-García G. , Psychometric Properties of the Spanish Version of the Edinburgh Handedness Inventory in a Sample of Chilean Undergraduates, Brain and Cognition. (2019) 137, 10.1016/j.bandc.2019.103618, 103618. 31629000

[35] Hagströmer M. , Oja P. , and Sjöström M. , The International Physical Activity Questionnaire (IPAQ): A Study of Concurrent and Construct Validity, Public Health Nutrition. (2006) 9, no. 6, 755–762, 10.1079/PHN2005898. 16925881

[36] Urbaniak G. C. and Plous S. , Research Randomize, 2013.

[37] Hardwick R. M. , Forrence A. D. , Krakauer J. W. , and Haith A. M. , Time-Dependent Competition Between Goal-Directed and Habitual Response Preparation, Nature Human Behaviour. (2019) 3, no. 12, 1252–1262, 10.1038/s41562-019-0725-0. 31570762

[38] Ciechanski P. and Kirton A. , Transcranial Direct-Current Stimulation can Enhance Motor Learning in Children, Cerebral Cortex. (2016) 10.1093/cercor/bhw114, bhw114. 27166171

[39] Sivaramakrishnan A. , Tahara-Eckl L. , and Madhavan S. , Spatial Localization and Distribution of the TMS-Related ‘Hotspot’ of the Tibialis Anterior Muscle Representation in the Healthy and Post-Stroke Motor Cortex, Neuroscience Letters. (2016) 627, 30–35, 10.1016/j.neulet.2016.05.041. 27222378 PMC5111166

[40] Fitzgerald P. B. and Daskalakis Z. J. , RTMS Treatment for Depression: A Practical Guide, 2022, Springer International Publishing, 10.1007/978-3-030-91519-3.

[41] Meehan S. K. , Dao E. , Linsdell M. A. , and Boyd L. A. , Continuous Theta Burst Stimulation Over the Contralesional Sensory and Motor Cortex Enhances Motor Learning Post-Stroke, Neuroscience Letters. (2011) 500, no. 1, 26–30, 10.1016/j.neulet.2011.05.237. 21683125

[42] Pleger B. , Blankenburg F. , and Bestmann S. , et al.Repetitive Transcranial Magnetic Stimulation-Induced Changes in Sensorimotor Coupling Parallel Improvements of Somatosensation in Humans, The Journal of Neuroscience. (2006) 26, no. 7, 1945–1952, 10.1523/JNEUROSCI.4097-05.2006. 16481426 PMC2635564

[43] Cai G. , Zhang C. , and Xu J. , et al.Efficacy of Transcranial Magnetic Stimulation in Post-Stroke Motor Recovery: Impact of Impairment Severity, IEEE Transactions on Neural Systems and Rehabilitation Engineering. (2025) 33, 881–889, 10.1109/TNSRE.2025.3543859. 40031445

[44] Tiffin J. and Asher E. J. , The Purdue Pegboard: Norms and Studies of Reliability and Validity, Journal of Applied Psychology. (1948) 32, no. 3, 234–247, 10.1037/h0061266. 18867059

[45] Podell K. , Kreutzer J. , DeLuca J. , and Caplan B. , Purdue Pegboard, Encyclopedia of Clinical Neuropsychology, 2017, Springer International Publishing, 1–3, 10.1007/978-3-319-56782-2_207-2.

[46] Vleugels L. W. E. , Swinnen S. P. , and Hardwick R. M. , Skill Acquisition is Enhanced by Reducing Trial-to-Trial Repetition, Journal of Neurophysiology. (2020) 123, no. 4, 1460–1471, 10.1152/jn.00741.2019. 32049588 PMC7191519

[47] Lai K. and Kelley K. , Accuracy in Parameter Estimation for ANCOVA and ANOVA Contrasts: Sample Size Planning via Narrow Confidence Intervals, British Journal of Mathematical and Statistical Psychology. (2012) 65, no. 2, 350–370, 10.1111/j.2044-8317.2011.02029.x. 22004142

[48] Pixa N. H. , Steinberg F. , and Doppelmayr M. , High-Definition Transcranial Direct Current Stimulation to Both Primary Motor Cortices Improves Unimanual and Bimanual Dexterity, Neuroscience Letters. (2017) 643, 84–88, 10.1016/j.neulet.2017.02.033. 28229937

[49] Walters S. J. , Jacques R. M. , Dos Anjos Henriques-Cadby I. B. , Candlish J. , Totton N. , and Xian M. T. S. , Sample Size Estimation for Randomised Controlled Trials With Repeated Assessment of Patient-Reported Outcomes: What Correlation Between Baseline and Follow-Up Outcomes Should we Assume?, Trials. (2019) 20, no. 1, 10.1186/s13063-019-3732-6. PMC6743178 31519202

[50] R. Core Team , R: A Language and Environment for Statistical Computing, 2025, R Foundation for Statistical Computing, Vienna, Austria.

[51] Wood S. N. , Generalized Additive Models: An Introduction With R, 2017, 2nd edition, Chapman and Hall/CRC, 10.1201/9781315370279.

[52] Moreno-Verdú M. , Waltzing B. M. , McAteer S. M. , Van Caenegem E. E. , Hamoline G. , and Hardwick R. M. , Information Processing in the Hand Laterality Judgement Task: Fundamental Differences Between Dorsal and Palmar Views Revealed by a “Forced Response” Paradigm, Cortex. (2025) 189, 226–241, 10.1016/j.cortex.2025.06.002. 40554923

[53] Pedersen E. J. , Miller D. L. , Simpson G. L. , and Ross N. , Hierarchical Generalized Additive Models in Ecology: An Introduction With Mgcv, PeerJ. (2019) 7, 10.7717/peerj.6876. PMC6542350 31179172

[54] Wood S. N. , Thin Plate Regression Splines, Journal of the Royal Statistical Society Series B: Statistical Methodology. (2003) 65, no. 1, 95–114, 10.1111/1467-9868.00374.

[55] Yong J. , MacDermid J. C. , and Packham T. , Defining Dexterity—Untangling the Discourse in Clinical Practice, Journal of Hand Therapy. (2020) 33, no. 4, 517–519, 10.1016/j.jht.2019.11.001. 31956020

[56] Saeed M. , Alfarra M. B. Q. , and Abdelmagied M. H. , et al.Comparative Analysis of Manual Dexterity of Dental Students at Ajman University Following One Academic Year of Preclinical Training Sessions: A Longitudinal Cohort Study, European Journal of Dentistry. (2023) 17, no. 4, 1179–1188, 10.1055/s-0042-1758793. 36535660 PMC10756829

[57] Sun R. R. , Wang Y. , and Fast A. , et al.Influence of Musical Background on Surgical Skills Acquisition, Surgery. (2021) 170, no. 1, 75–80, 10.1016/j.surg.2021.01.013. 33608147

[58] Yoshitake Y. , Ikeda A. , and Shinohara M. , Robotic Finger Perturbation Training Improves Finger Postural Steadiness and Hand Dexterity, Journal of Electromyography and Kinesiology. (2018) 38, 208–214, 10.1016/j.jelekin.2017.11.011. 29199081

[59] Ohashi H. , Gribble P. L. , and Ostry D. J. , Somatosensory Cortical Excitability Changes Precede Those in Motor Cortex During Human Motor Learning, Journal of Neurophysiology. (2019) 122, no. 4, 1397–1405, 10.1152/jn.00383.2019. 31390294 PMC6843109

[60] Kumar N. , Manning T. F. , Ostry D. J. , and Ganguly K. , Somatosensory Cortex Participates in the Consolidation of Human Motor Memory, PLoS Biology. (2019) 17, no. 10, 10.1371/journal.pbio.3000469. PMC6793938 31613874

[61] Choi E.-H. , Yoo W.-K. , Ohn S. H. , Ahn S. , Kim H. J. , and Jung K.-I. , Enhancement of Motor Coordination by Applying High Frequency Repetitive TMS on the Sensory Cortex, Journal of Electromyography and Kinesiology. (2016) 28, 17–22, 10.1016/j.jelekin.2016.02.008. 26978587

[62] Brodie S. M. , Meehan S. , Borich M. R. , and Boyd L. A. , 5 Hz Repetitive Transcranial Magnetic Stimulation Over the Ipsilesional Sensory Cortex Enhances Motor Learning After Stroke, Frontiers in Human Neuroscience. (2014) 8, 10.3389/fnhum.2014.00143. PMC3968757 24711790

[63] Gilio F. , Iacovelli E. , and Frasca V. , et al.Electrical and Magnetic Repetitive Transcranial Stimulation of the Primary Motor Cortex in Healthy Subjects, Neuroscience Letters. (2009) 455, no. 1, 1–3, 10.1016/j.neulet.2009.03.035. 19429094

[64] Wessel M. J. , Beanato E. , and Popa T. , et al.Noninvasive Theta-Burst Stimulation of the Human Striatum Enhances Striatal Activity and Motor Skill Learning, Nature Neuroscience. (2023) 26, no. 11, 2005–2016, 10.1038/s41593-023-01457-7. 37857774 PMC10620076

[65] Khanmohammadi S. , Ehsani F. , Bagheri R. , and Jaberzadeh S. , Compared Motor Learning Effects of Motor Cortical and Cerebellar Repetitive Transcranial Magnetic Stimulation During a Serial Reaction Time Task in Older Adults, Scientific Reports. (2025) 15, no. 1, 10.1038/s41598-025-95859-1, 12447. 40216873 PMC11992138

[66] Nojima I. , Watanabe T. , Gyoda T. , Sugata H. , Ikeda T. , and Mima T. , Transcranial Static Magnetic Stimulation Over the Primary Motor Cortex Alters Sequential Implicit Motor Learning, Neuroscience Letters. (2019) 696, 33–37, 10.1016/j.neulet.2018.12.010. 30552943

[67] Rumpf J.-J. , May L. , Fricke C. , Classen J. , and Hartwigsen G. , Interleaving Motor Sequence Training With High-Frequency Repetitive Transcranial Magnetic Stimulation Facilitates Consolidation, Cerebral Cortex. (2020) 30, no. 3, 1030–1039, 10.1093/cercor/bhz145. 31373620 PMC7132921

[68] Weiler F. , Brandão P. , Barros-Filho J. D. , Uribe C. E. , Pessoa V. F. , and Brasil-Neto J. P. , Low Frequency (0.5 Hz) rTMS Over the Right (Non-Dominant) Motor Cortex Does not Affect Ipsilateral Hand Performance in Healthy Humans, Arquivos de Neuro-Psiquiatria. (2008) 66, no. 3b, 636–640, 10.1590/S0004-282X2008000500006. 18949254

[69] Jwa A. S. , Goodman J. S. , and Glover G. H. , Inconsistencies in Mapping Current Distribution in Transcranial Direct Current Stimulation, Frontiers in Neuroimaging. (2023) 1, 10.3389/fnimg.2022.1069500, 1069500. 37555148 PMC10406311

[70] Datta A. , Elwassif M. , and Bikson M. , Bio-Heat Transfer Model of Transcranial DC Stimulation: Comparison of Conventional Pad Versus Ring Electrode, *Annual International Conference of the IEEE Engineering in Medicine and Biology Society*, 2009, Minneapolis, MN, IEEE, 670–673, 10.1109/IEMBS.2009.5333673. 19964238

[71] Priori A. , Hallett M. , and Rothwell J. C. , Repetitive Transcranial Magnetic Stimulation or Transcranial Direct Current Stimulation?, Brain Stimulation. (2009) 2, no. 4, 241–245, 10.1016/j.brs.2009.02.004. 20633424

[72] Kesikburun S. , Non-Invasive Brain Stimulation in Rehabilitation, Turkish Journal of Physical Medicine and Rehabilitation. (2022) 68, no. 1, 1–8, 10.5606/tftrd.2022.10608. 35949977 PMC9305642

[73] Schuelke M. J. and Day E. A. , Seel N. M. , Ability Determinants of Complex Skill Acquisition, Encyclopedia of the Sciences of Learning, 2012, Springer US, 20–23, 10.1007/978-1-4419-1428-6_798.

[74] Ebrahimi S. , Van Der Voort B. , and Ostry D. J. , The Consolidation of Newly Learned Movements Depends Upon the Somatosensory Cortex in Humans, The Journal of Neuroscience. (2024) 44, no. 32, 10.1523/JNEUROSCI.0629-24.2024. PMC11308319 38871461

[75] Bhattacharjee S. , Kashyap R. , Abualait T. , Annabel Chen S.-H. , Yoo W.-K. , and Bashir S. , The Role of Primary Motor Cortex: More Than Movement Execution, Journal of Motor Behavior. (2021) 53, no. 2, 258–274, 10.1080/00222895.2020.1738992. 32194004

[76] Shelchkova N. D. , Downey J. E. , and Greenspon C. M. , et al.Microstimulation of Human Somatosensory Cortex Evokes Task-Dependent, Spatially Patterned Responses in Motor Cortex, Nature Communications. (2023) 14, no. 1, 10.1038/s41467-023-43140-2, 7270. PMC10638421 37949923

[77] Sansare A. , Magalhaes T. N. C. , and Bernard J. A. , Relationships Between Balance Performance and Connectivity of Motor Cortex With Primary Somatosensory Cortex and Cerebellum in Middle Aged and Older Adults, 2024, 147, bioRxiv, 1–11, 10.1101/2024.03.29.587335. PMC11973825 39637518

[78] Psurek F. , King B. R. , Classen J. , and Rumpf J.-J. , Offline Low-Frequency rTMS of the Primary and Premotor Cortices Does not Impact Motor Sequence Memory Consolidation Despite Modulation of Corticospinal Excitability, Scientific Reports. (2021) 11, no. 1, 10.1038/s41598-021-03737-3, 24186. 34921224 PMC8683442

[79] Yin H. H. and Knowlton B. J. , The Role of the Basal Ganglia in Habit Formation, Nature Reviews Neuroscience. (2006) 7, no. 6, 464–476, 10.1038/nrn1919. 16715055

[80] Derosiere G. , Shokur S. , and Vassiliadis P. , Reward Signals in the Motor Cortex: From Biology to Neurotechnology, Nature Communications. (2025) 16, no. 1, 10.1038/s41467-024-55016-0, 1307. PMC11791067 39900901

[81] Molina-Luna K. , Pekanovic A. , and Röhrich S. , et al.Dopamine in Motor Cortex is Necessary for Skill Learning and Synaptic Plasticity, PLoS ONE. (2009) 4, no. 9, 10.1371/journal.pone.0007082. PMC2738964 19759902

[82] Rioult-Pedotti M.-S. , Pekanovic A. , Atiemo C. O. , Marshall J. , and Luft A. R. , Dopamine Promotes Motor Cortex Plasticity and Motor Skill Learning via PLC Activation, PLoS ONE. (2015) 10, no. 5, 10.1371/journal.pone.0124986. PMC4418826 25938462

[83] Mawase F. , Uehara S. , Bastian A. J. , and Celnik P. , Motor Learning Enhances Use-Dependent Plasticity, The Journal of Neuroscience. (2017) 37, no. 10, 2673–2685, 10.1523/JNEUROSCI.3303-16.2017. 28143961 PMC5354321

[84] Ramakrishnan A. , Byun Y. W. , Rand K. , Pedersen C. E. , Lebedev M. A. , and Nicolelis M. A. L. , Cortical Neurons Multiplex Reward-Related Signals Along With Sensory and Motor Information, Proceedings of the National Academy of Sciences. (2017) 114, no. 24, 10.1073/pnas.1703668114. PMC5474796 28559307

[85] Krakauer J. W. , Hadjiosif A. M. , Xu J. , Wong A. L. , and Haith A. M. , Terjung R. , Motor Learning, Comprehensive Physiology, 2019, 1st edition, Wiley, 613–663, 10.1002/cphy.c170043. 30873583

[86] Kim Y.-H. , Park J.-W. , Ko M.-H. , Jang S. H. , and Lee P. K. W. , Facilitative Effect of High Frequency Subthreshold Repetitive Transcranial Magnetic Stimulation on Complex Sequential Motor Learning in Humans, Neuroscience Letters. (2004) 367, no. 2, 181–185, 10.1016/j.neulet.2004.05.113. 15331148

[87] Valero-Cabré A. , Pascual-Leone A. , and Rushmore R. J. , Cumulative Sessions of Repetitive Transcranial Magnetic Stimulation (rTMS) Build Up Facilitation to Subsequent TMS-Mediated Behavioural Disruptions, European Journal of Neuroscience. (2008) 27, no. 3, 765–774, 10.1111/j.1460-9568.2008.06045.x. 18279329

[88] Hallett M. , Transcranial Magnetic Stimulation: A Primer, Neuron. (2007) 55, no. 2, 187–199, 10.1016/j.neuron.2007.06.026. 17640522

[89] Karabanov A. , Ziemann U. , and Hamada M. , et al.Consensus Paper: Probing Homeostatic Plasticity of Human Cortex With Non-Invasive Transcranial Brain Stimulation, Brain Stimulation. (2015) 8, no. 5, 993–1006, 10.1016/j.brs.2015.06.017. 26598772

[90] Holmes N. P. , Tamè L. , and Beeching P. , et al.Locating Primary Somatosensory Cortex in Human Brain Stimulation Studies: Experimental Evidence, Journal of Neurophysiology. (2019) 121, no. 1, 336–344, 10.1152/jn.00641.2018. 30575432 PMC6383658

