# Supplementary material for: Effects of Different Repetitive Transcranial Magnetic Stimulation Targets for Enhancing Motor Learning: A Single‐Blind Randomized Controlled Trial
Source: Neural Plast. 2026 Jun 4;2026:6487874. doi: 10.1155/np/6487874 (PMC13581060; doi:10.1155/np/6487874)
Supplement: Supplementary file 1 [file NP-2026-6487874-s001.docx]

**Supplementary Materials**

**Manipulation check: Statistical analysis of training data across groups**

As a fundamental manipulation check, this analysis consisted of confirming that participants generally improved in performance in the two tasks they were trained in, regardless of the rTMS protocol received. These analyses therefore ignored treatment assignment (that is, no between-group comparisons were performed), as we were interested only in the crude effect of training. As such, we considered only the data collected during the training sessions (both PPT and AVMA), not the formal assessments. The models below were Generalized Linear Mixed Models (GLMMs) with by-participant random intercepts and slopes (wherever possible), to account for individual differences in learning across sessions.

Purdue Pegboard Test (PPT):

- Number of pegs (in 30 seconds): For simplicity, we fitted a LMM with a Gaussian distribution for this variable. Again, Session was scaled and centred and included as a numeric predictor, and the type of PPT was included as a categorical predictor (Left hand, Right hand or Both hands) that interacted with Session, by the formula: *Pegs ~ Session * Type + (Session | ID)*. We note we did not include the factor Type as a random slope as it caused singularity issues, therefore we simplified the model to include only Session as random slope as we expected this was going to be main driver of the effects. Again, the effect size was calculated as the slope for the fixed effect of Session, obtained from the model predictions. In this case a separate slope was obtained for each Type of PPT performed.

Rapid response AVMA task:

- Reaction time (ms): The data was trimmed to exclude trials with extremely short (<100ms) or long (>1,500ms), which removed 1686 trials out of 311,154 (representing 0.54%). On the trimmed dataset, we fitted a GLMM with a Gamma distribution, as reaction time was right-skewed. To improve convergence, the numeric predictor Session was scaled and centred, by the formula: *Reaction time ~ Session + (Session | ID)*. The effect size was calculated as the slope for the fixed effect of Session, obtained from the model predictions on the response scale (back-transformed from the log scale).
- Errors: The data was first trimmed as in the model above. We aggregated trials into blocks of 100 trials per participant per session, computing the number of correct and incorrect responses in each block. To account for the binomial nature of accuracy data and the highly skewed distribution of errors, we fitted a GLMM with a binomial distribution, using the formula: *cbind(n_correct, n_errors) ~ Session + (Session | ID)*. The numeric predictor Session was scaled and centred and included as a random slope for participant. The effect size was expressed as the estimated slope of Session on the expected probability of making an error per block of 100 trials, obtained from the model predictions on the response scale (back-transformed from the logit scale).

**Results of manipulation checks**

**Supplementary Fig. 1.** Results of the training analysis for the Purdue Pegboard Test (PPT) throughout training. Across sessions, all three outcome measures improved with training.

**Supplementary Fig. 2.** Results of the training analysis for the rapid response Arbitrary VisuoMotor Association (AVMA) task. **Panel a** shows reaction time and **Panel b** shows number of errors per training session. Across sessions, both measures improved with training.

**Baseline assessment in AVMA**

We ran a separate GAMM considering the data for the baseline assessment (t0) to confirm that before the intervention, there were no differences between the groups in the AVMA. We did this instead of including the baseline performance as a covariate in the post-training GAMMs, given the impossibility to do so as in the AVMA there is no ‘unique’ value of performance, because the whole speed-accuracy trade-off function is computed across preparation time.

The results showed that the speed-accuracy trade-offs were comparable between groups at t0 (Fig. S3a), with no statistically significant differences in any of the comparisons after correcting for multiple comparisons (Fig. S3b-c).

**Supplementary Fig. 3.** Results of the Forced Response Arbitrary Visuomotor Association (FR-AVMA) task at baseline (t0). **Panel a** shows the model-based speed-accuracy trade-off function for each group. **Panels b-d** show the differences in the proportion of correct responses between groups. The line becomes red where the 95% confidence interval does not overlap with 0. However, the p-values were never < 0.05 after correcting for multiple comparisons by the False Discovery Rate.
